# Fieldable Microfluidic Platform for Separation and Assay of U and Pu from Fission Samples in Environmental Matrices

**DOI:** 10.1021/acs.iecr.6c01280

**Published:** 2026-07-06

**Authors:** Kevin J. Glennon, Hector F. Valdovinos, Jake A. Bence, Tashi Parsons-Davis, Narek Gharibyan, Jennifer A. Shusterman

**Affiliations:** Nuclear and Chemical Sciences Division, 4578 Lawrence Livermore National Laboratory, Livermore, California 94550, United States

## Abstract

An integrated microfluidic platform has been designed for in-field separation and assay of Pu and U from environmental samples. Separation of Pu and U from the sample matrix is accomplished by performing two sequential liquid–liquid extractions using 3D-printed supported liquid membrane (SLM) modules on the microfluidic scale. The initial extraction separates Pu and U from the bulk sample matrix, and the following extraction separates Pu from U for individual isotopic assay of the elements. Tested sample matrices were dissolved NIST SRM 2706 (New Jersey Soil), dissolved NIST SRM 365a (Portland Cement), and undiluted Atlantic Seawater. Chemistry is complete within 1 h of initializing the platform, and isotopic measurements from these matrices have been benchmarked with standardized sources. Typical yields are >90% and >40% for U and Pu, respectively. Generally, <1% of most fission product elements coextract with either U or Pu.

## Graphical abstract

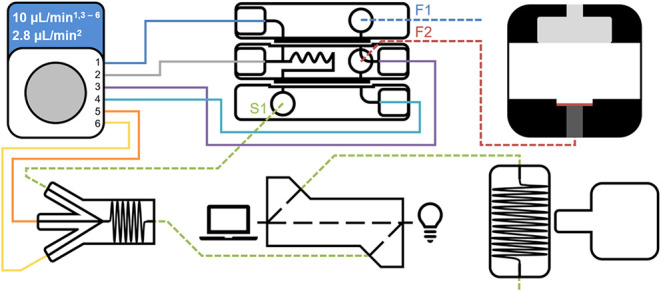

## 1 Introduction

Postdetonation nuclear forensic analysis involves radiochemical measurements of debris from a nuclear event targeting analytes across most of the periodic table.^1–4^ Thorough measurements of actinide, fission product (FP), and activation product (AP) inventories in such debris can provide technical information to support prosecution and attribution efforts.^1,5,6^ The chemical separations required for these measurements are complicated by the wide range of matrix elements (MEs) from the surrounding environment which are incorporated into the debris.^7–10^

Developing fieldable radiochemical separations and assays could expedite preliminary analyses, starting as soon as samples are collected. Deploying entire radiochemistry laboratories to the field is not ideal, as analytical procedures utilizing column chromatography, precipitations, and liquid–liquid extractions often require large amounts of hazardous reagents, space, and specialized instrumentation for performing the analyses.^1,3,4,11^ However, a microfluidic chemistry platform may be suitable for performing a subset of optimized radiochemical procedures in the field, as microfluidic technologies have small physical footprints, minimize the quantity of reagents used, have potential for automation, and provide equal or greater separation factors as traditional techniques.^12–20^

Previous works by the authors have reported the development of a 3D printed flat-sheet supported liquid membrane (FS-SLM) microfluidic device capable of extracting U­(VI) from HNO_3_ using 30% tributylphosphate (TBP) in *n*-dodecane,^19^ a Pu­(III)/U­(VI) separation utilizing an improved FS-SLM platform followed by an online isotopic analysis of the ^238^Pu/^239,240^Pu activity ratio using alpha spectrometry,^5^ and an online measurement of the ^237^U/^total^U specific activity of the U product from the FS-SLM platform using a UV–vis flow cell and a small CdTe gamma spectrometer.^6^ The prior works were constrained to use exclusively fieldable technologies determined by size, reagent hazards and stability, and power requirements. The integrated system described here requires <1,500 W peak power and <24 ft^2^ table space using portable instrumentation, electronics, and peristaltic pumps. This work expands upon the previous by combining multiple FS-SLM extractions into a single process flow using a newly developed “Extraction Tower” design, enabling Pu­(IV) and U­(VI) to be coseparated from a sample matrix followed by a Pu­(III)/U­(VI) separation. The individual U and Pu measurement platforms have been integrated to execute both measurements from a single sample. In addition, this work reports U and Pu extraction yields when challenged with samples containing various environmental matrices of soil, cement, and seawater—along with Np and FP extraction yields from nonmatrixed samples. Finally, the fully integrated system is tested by performing end-to-end separations and analyses of U and Pu from surrogate debris samples containing short-lived FPs in environmental matrices and benchmarking the measured values against standardized values. The experiments and validations reported here were performed in a laboratory environment, as required before the platform may be deployed in a fielded condition.

This FS-SLM platform has been designed for rapid analysis of postdetonation nuclear debris using a 30% TBP extractant, but as a technology its applications may be broader. A fieldable U and Pu extraction and analysis platform could be directly applied to swipe and environmental samples taken at or near nuclear facilities for safeguards applications,^21,22^ without having to send samples back to an established laboratory. The extractant may be changed based on intended application; by adjusting the extractant, the platform could also be developed as an analytical tool for other metals which require extraction from environmental matrices.^23^ Microfluidic platforms that can selectively extract metals from environmental samples for fieldable detection have been specifically identified as ideal techniques for a range of critical analyses.^24,25^

## 2 Methods

The Formlabs Form 4 and stereolithography (SLA) 3D printers and Clear V4 and V5 resins were acquired from Formlabs (MA, USA). All nitric acid was used as trace-element grade from Fisher Scientific (NH, USA). TBP was purchased as 97% from Millipore Sigma (MA, USA), and *n*-dodecane was used as 99% from Tokyo Chemical Industries (Tokyo, Japan). All TBP was washed with sodium carbonate and pre-equilibrated with an equal volume of 3 M HNO_3_ prior to use. Ascorbic acid was obtained as >99% ACS grade from Fisher Chemical and sulfamic acid as ACS grade from EMD Chemicals Inc. The 25 mm diameter, 80 μm thick hydrophobic PTFE membrane with 0.2 μm pore size was purchased from Advantec MFS, Inc. (CA, USA). ^238^Pu and ^233^U were obtained as legacy material from Lawrence Livermore National Laboratory (LLNL, CA, USA). The certified reference material (CRM) 138 Pu isotopic standard was obtained from NNSA Nuclear Reference Material Program, formerly New Brunswick Laboratory Program Office (TN, USA). ^238^Pu and CRM 138 Pu were used as purified by anion exchange column chromatography. The CRM 138 Pu was loaded in 8 M HNO_3_ onto a column packed with Eichrom AG 1-X4 anion exchange resin. The column was washed with 10 column volumes (CVs) 8 M HNO_3_ and 5 CVs 10 M HCl prior to elution of Pu with 7.5 CVs 10:1 v:v 10 M HCL: conc. HI. The Pu was converted to nitrate by evaporating several times with concentrated HNO_3_, then dissolved in 4 M HNO_3_ with 0.008 M HF. A 1,000 ppm U inductively coupled plasma mass spectrometry (ICP-MS) standard was obtained from Inorganic Ventures (VA, USA). Isopropyl alcohol was purchased from VWR BDH Chemicals (PA, USA). All H_2_O was purified using a Milli-Q 18.2 MΩ cm purification system purchased from Millipore Sigma (MA, USA).

Fluorinated ethylene-propylene (FEP) tubing, 1/4″-28 flangeless Delrin nuts, and ETFE flangeless ferrules were acquired from Idex Health & Science (WA, USA). All O-rings were obtained from McMaster-Carr (IL, USA). All alpha spectrometry was performed with a Canberra PIPS 600 mm^2^ silicon detector using an Ortec 428 bias supply, an 855 Dual Spec amplifier, and a 927 ASPEC multichannel analyzer (MCA) powered by an Ortec 4006 minibin power supply and evacuated with the 17 W ALPHA-MINI-PPS vacuum pump. The 1 cm flow cell was acquired from FIAlab Instruments (WA, USA) and the UV–visible absorbance spectrophotometry Flame-S was acquired from Ocean Insight (FL, USA). The portable gamma-ray spectrometry model X-123 CdTe was purchased from Amptek (MA, USA). The 4-channel independently controlled (ICC) Reglo peristaltic pump was purchased from Ismatec through Cole-Parmer (IL, USA).

### 2.1 Sample Preparations

A variety of samples were prepared to validate the platform as described. Neat samples individually containing ^233^U and CRM 138 Pu at approximately 170 Bq in 1.2 mL 3 M HNO_3_ were prepared for the steady state and flow rate studies. Matrixed samples were prepared by individually adding ^233^U and CRM 138 Pu to previously dissolved soil, cement, and seawaters standards for the matrix study. Surrogate debris samples containing ^237^U, ^239^Np, and mixed fission products in 3 M HNO_3_ were used for extraction yield studies of various short-lived FPs and Np. Finally, a set of 5 samples containing U and Pu in different environmental matrices were prepared to benchmark the fully integrated system against a subset of known U and Pu isotopics. This section describes the preparation of each sample set in detail. All FS-SLM extractions using any FPs were performed within 1 week of FP production. CRM 138 Pu was the only radionuclide standard used for any of these samples. The certified ^238^Pu/^239,240^Pu activity ratio was decay-corrected from the CRM 138 certificate to (1.74 ± 0.17) × 10^–2^; due to the relatively high uncertainty of the certified ^238^Pu value, the ratio was separately standardized using alpha spectrometry of a neat sample as (1.81 ± 0.02) × 10^–2^ as of February 2023 when the experiments were performed. These certified and standardized ratios are reported at 1σ uncertainty, and methods for determining standardized values are discussed in the SI.

The feed solutions used in the matrix study were prepared by adding approximately 170 Bq ^233^U and CRM 138 Pu radiotracers to individually prepared 1.2 mL aliquots of each dissolved matrix solution then evaporating to dryness and reconstituting in 1.2 mL of 3 M HNO_3_ twice. Three matrix standards were tested as part of this work: 1) SRM 2706 New Jersey Soil, 2) SRM 635a Portland Cement, and 3) Atlantic Seawater working standard (Ocean Scientific International Ltd.). The soil and cement matrices were each dissolved into homogeneous solutions by heating to dryness with several cycles of conc. HNO_3_, conc. HCl, and aqua regia with <2 M HF in <10 mL increments. The soil matrix was digested to a final solution of 56.6 mg SRM 2706 per mL in conc. HNO_3_. The cement matrix was digested to a final solution of 24.5 mg SRM 635a per mL in 6 M HCl. The seawater matrix was used as received. Each matrix was screened by inductively coupled plasma mass spectrometry (ICP-MS) as prepared for extraction to determine the concentrations of most elements specified on the corresponding standard certificates. This analysis is represented in Figure 1 and tabulated in the SI as Table S1 along with the associated ICP-MS analytical methods. Some elements, such as silicon and chlorine, were reduced or removed from solution as part of the dissolution process with HF and HNO_3_.

**Figure 1:**
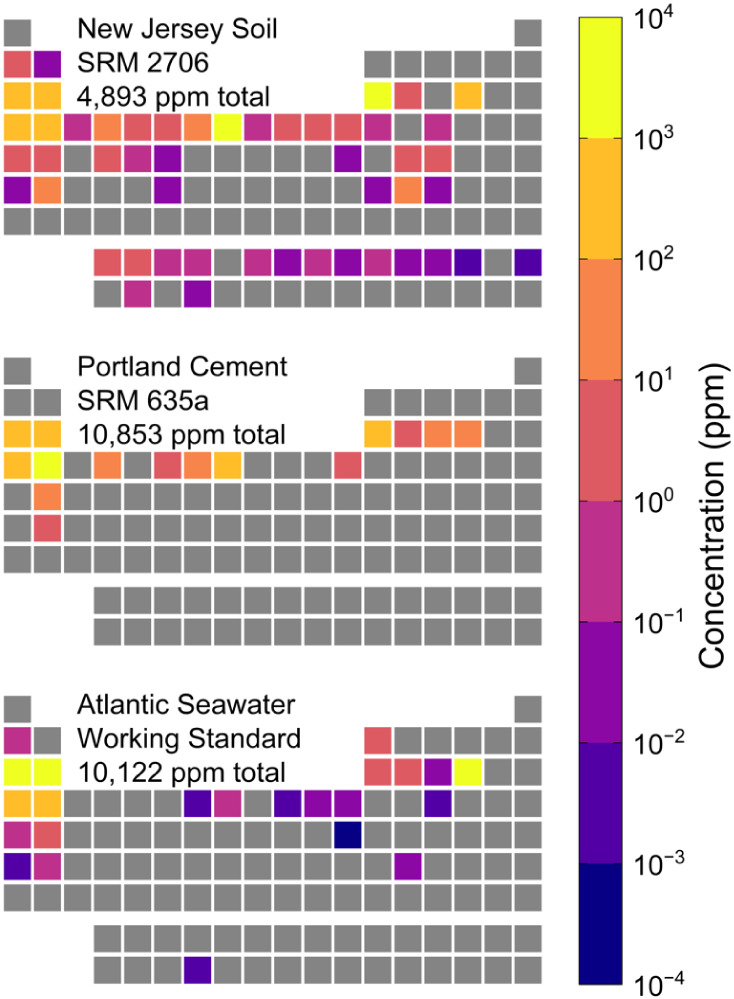
Trace element analysis. Element concentrations assayed via ICP-MS in the three matrix solutions as prepared for extraction in 3 M HNO_3_.

The following U, Np, and FP fractions were used to prepare surrogate debris samples in 3 M HNO_3_, each containing approximately 10^12^ fissions, 7 × 10^4^ Bq ^239^Np, and 5 × 10^2^ Bq ^237^U in 1.2 mL 3 M HNO_3_ for the extraction yield study. Multiple FP and ^237^U sources were produced by irradiating approximately 250 mg cm^–2^ of ultradepleted ^238^U metal foil with 15 MeV protons at the Center for Accelerated Mass Spectrometry (CAMS) facility at LLNL, producing approximately 10^13^ fissions and 2.2 × 10^5^ Bq ^237^U (1.5 × 10^6^ Bq/g) per irradiation. Exclusively for sample preparation, the U was separated from FPs using extraction chromatography such that it could be added back in at appropriate concentrations for testing the FS-SLM platform. The irradiated foils were dissolved while heating in 2 mL of 6 M HCl with 3 drops of 8 M HNO_3_, dried down, and reconstituted in 6 M HNO_3_ and loaded onto 1 cm inner diameter (ID) polyethylene columns filled with LN resin (Eichrom) up to a bed height of 4.75 cm. FPs were eluted from the column with an additional 9 mL 6 M HNO_3_, 6 mL 9 M HNO_3_, 8 mL 0.5 M HCl–2% H_2_O_2_, and 6 mL 0.1 M HNO_3_–0.1 M HF. U was eluted into its own fraction using 8 mL 9 M HCl. The separated U fraction was assayed for ^237^U activity and ^total^U concentration using gamma spectrometry with high purity germanium (HPGe) detectors and ICP-MS, respectively; these assays are described in the SI and were used to determine the known U isotopics of each surrogate fission sample used. ^239^Np tracer was isolated from a depleted uranium target that had been irradiated with thermal neutrons at the McClellan Nuclear Research Center (MNRC) at University of California, Davis. The target was dissolved in 9 M HCl with trace HNO_3_ then converted to 4 M HNO_3_. Using iron­(II) sulfamate as a reducing agent, the Np was purified by lanthanum fluoride precipitation followed by two rounds of anion exchange column chromatography. After several cycles of evaporating in conc. HNO_3_, the eluted Np was dissolved in 3 M HNO_3_.

Five samples were prepared to benchmark the integrated system. Three of the samples were prepared by adding 300 μg of the proton-irradiated U and 0.0300 μg of CRM 138 Pu to 10^12^ fissions in 1.2 mL 3 M HNO_3_ containing the soil, cement, and seawater matrix solutions, producing a ^total^U concentration of 250 ppm. Two additional samples were prepared without environmental matrices (in 3 M HNO_3_) at 250 and 4 ppm U. The ^237^U/^total^U specific activity in all five samples was standardized to be (1.521 ± 0.037) × 10^6^ Bq/g using the methods described in the SI.

### 2.2 Microfluidic FS-SLM Extraction Procedures

The microfluidic modules were printed using a Formlabs Form 4 printer with Clear V5 resin at a layer height of 50 μm. The SI describes the three different modules in detail: the Initiator, the Propagator, and the Terminator, also represented as Figure 2i, 2ii, and 2iii. Coupling an Initiator module directly to a Terminator module results in a system capable of performing a single extraction, as demonstrated in previous publications by our group. A Propagator module was developed for inclusion between the Initiator and Terminator modules, producing an Extraction Tower capable of performing multiple sequential extractions. The Extraction Tower was designed to include *n* Propagator modules to perform *n* + 1 sequential extractions; in this work, the *n* = 1 Extraction Tower was sufficient for the separation process investigated, capable of performing two sequential extractions.

**Figure 2:**
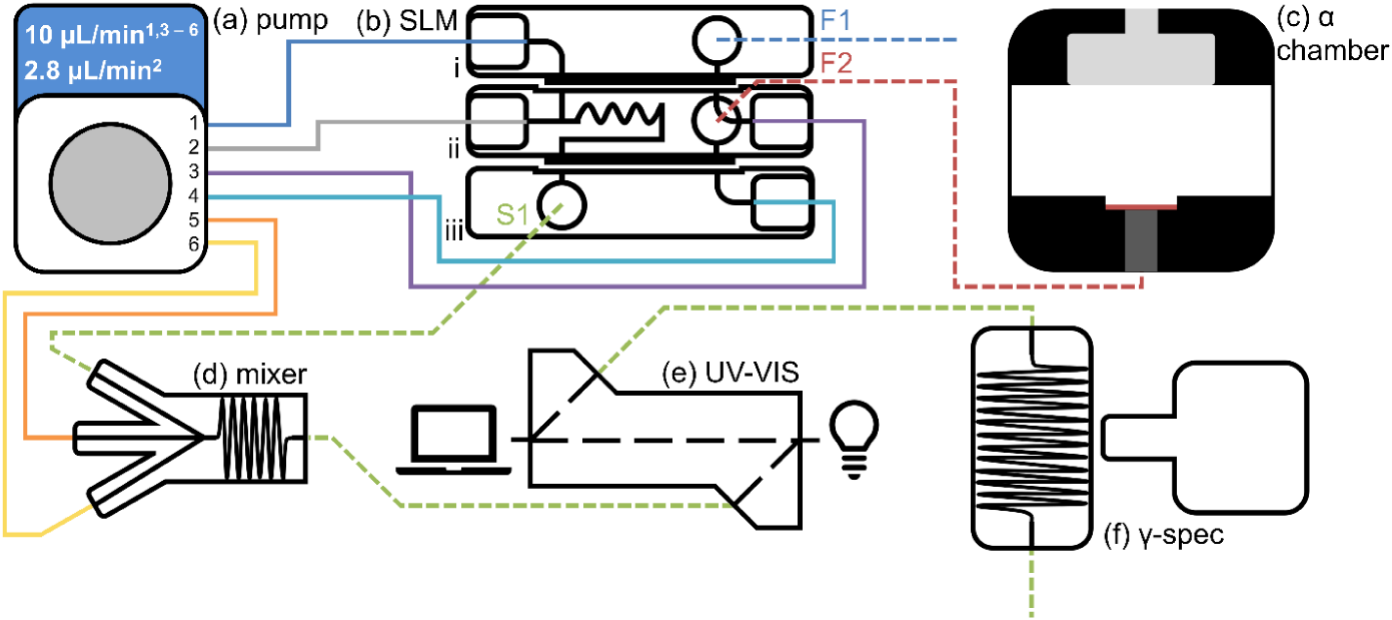
Schematic of the fully integrated FS-SLM platform. (a) Two 4-channel peristaltic pumps with independent channel control; (b) the Extraction Tower; (c) custom 3D printed alpha chamber; (d) 3D printed PAR (4-(2-pyridylazo)-resorcinol) mixer; (e) 1 cm flow cell, light source, and UV–vis detector; (f) 3D printed sample loop and CdTe detector. 1) sample feed in 3 M HNO_3_ at 10 μL min^–1^; 2) 13 M HNO_3_ at 2.8 μL min^–1^; 3) 0.3 M HNO_3_ with 1 mM ascorbic acid and 5 mM sulfamic acid at 10 μL min^–1^; 4) 0.3 M HNO_3_ at 10 μL min^–1^; 5) 36.3 mg/mL PAR in pH 8 1.3 M NH_4_OH/0.6 M H_3_BO_3_ (“PAR solution”) at 10 μL min^–1^; 6) U calibration standards at 10 μL min^–1^; (i) Initiator module; (ii) Propagator module; (iii) Terminator module; F1) Most actinides, FPs, and MEs outlet stream; F2) Pu outlet stream; S1) U outlet stream. Components are not drawn to scale.

System assembly, parts, instrumentation, and extraction procedures are detailed in previous publications.^5,6,19^ A schematic describing the fully integrated process flow, components, inlet streams, and outlet streams is provided as Figure 2. Briefly, 80 μm thick PTFE membranes are placed between the extraction channels of the Initiator, Terminator, and Propagator modules, then soaked in a 30% TBP in *n*-dodecane extractant to create a flat-sheet extraction membrane. Sample solutions are introduced in 3 M HNO_3_ over the topside of the first extraction membrane, which selectively extracts U­(VI) and Pu­(IV). A reducing strip solution of 0.3 M HNO_3_ with 1 mM ascorbic acid and 5 mM sulfamic acid is eluted across the bottom of the first membrane, stripping U­(VI) and Pu­(III). The reducing strip enters an internal mixing channel where it mixes with 13 M HNO_3_ at a low flow rate, concentrating up to 3 M HNO_3_. This stream flows across the top of the second TBP membrane which extracts U­(VI) but not Pu­(III). U­(VI) is stripped from the bottom of the second membrane using a stream of 0.3 M HNO_3_. Each outlet stream is collected into individual vials (yielding experiments) or eluted directly to instrumentation for assay (fully integrated system). In the *n* = 1 Extraction Tower, the F1 outlet stream is expected to carry most actinides, FPs, and MEs, the F2 outlet stream is expected to carry Pu­(III), and the S1 outlet stream is expected to carry U­(VI).

### 2.3 Extraction Yield Experiments

Yielding experiments were not performed with the fully integrated system; instead, each outlet stream was eluted into individual vials. U and Pu yields were measured from individual extractions by liquid scintillation counting (LSC), while FP and Np yields were measured from the same extractions by gamma spectrometry utilizing an HPGe. Mass balance and extraction yield were determined by weighing the mass of each outlet stream collected and the mass of the sample solution eluted through the Extraction Tower after steady state, then taking weighed aliquots of each outlet stream and the sample solution for assay. An example is provided for calculating yield in the F1 stream as eq 1, where A_LSC,F1_ is the alpha activity measured in an aliquot of the F1 stream by LSC, m_LSC,F1_ is the mass of the aliquot taken from the F1 stream for LSC analysis, m_F1_ is the total mass of the F1 stream collected, and m_Feed_ is the total mass of the feed solution eluted after steady state. Uncertainties are derived from nuclear decay counting statistics and are reported as the square root of the greater internal or external 1σ variance of the weighted average of duplicate or triplicate extractions.

1 YieldF1=(ALSC,F1mLSC,F1)mF1(ALSC,FeedmLSC,Feed)mFeed

The steady state and flow rate studies were performed as single extractions (without Propagator modules) in triplicate by varying either the steady state or flow rate parameters, while holding the other constant. The ideal flow rate was used directly for all further extractions, while the ideal steady state time (*t_S_*_0_) was extrapolated to the *n =* 1 Extraction Tower (*t_S_*_1_) as *t_S_*_1_ = *t_S_*_0_ * 2 + *V_D_*/*v* where *V_D_* is the dead volume in the assembled tower (Table S2), and *v* is the flow rate of the major streams. This steady state time was used for all yielding experiments performed with the Extraction Tower. The steady state and flow rate studies were performed with sample solutions of 3 M HNO_3_ and stripping solutions of 0.3 M HNO_3_ without ascorbic or sulfamic acids.

The *n* = 1 Extraction Tower was used for the matrix study to measure U and Pu yields in duplicate using both 1 mM ascorbic acid and 5 mM sulfamic acid in the reducing strip (Figure 2, stream 3). The Extraction Tower was also used for the decontamination study to measure short-lived FP and Np yields without sample matrix in triplicate. In most cases, the detection limit of each FP was limited by the large activity of ^239^Np present; to mitigate this, multiple gamma spectra were collected at different counting intervals to allow ^239^Np to decay and increase sensitivity to the longer-lived FPs. The decontamination study utilized 1 mM ascorbic acid without sulfamic acid in the reducing strip (Figure 2, stream 3).

### 2.4 The Integrated System for Fieldable U and Pu Isotopic Analysis

Our previous works should be referenced for detailed discussions of each isotopic analysis, including operating conditions, benchmarks against standardized values, and uncertainty quantifications.^5,6^ In each of the extractions performed here, U and Pu were separated from complex sample matrices using the *n* = 1 Extraction Tower, then individual online isotopic measurements were performed as described.

The U outlet stream is designated as S1 in Figure 2. The S1 stream was continuously eluted through a UV–vis flow cell and a 110 μL 3D-printed sample loop sitting at the face of a CdTe detector. In solutions with ^total^U < 10 ppm, the S1 stream was mixed with a buffered PAR (4-(2-pyridylazo)-resorcinol) solution (Figure 2d, stream 5, prepared shortly before use) prior to eluting through the 1 cm-long flow cell (SMA-Z-10, FIAlab Instruments). The UV–vis spectrometer (Flame-S, Ocean Insight) measured ^total^U concentration in real-time, confirming when steady state was achieved. After steady state, the CdTe sample loop was disconnected from the system and used to measure the activity of ^237^U per μL over an extending counting interval, up to 21 h. The ratio of these measurements is reported as the specific activity of ^237^U/^total^U in units of Bq/g.

The Pu outlet stream is designated as F2 from Figure 2. The F2 outlet stream was continuously eluted into a waste vial until the UV–vis spectrometer confirmed steady state had been achieved. Then, approximately 100 μL of the F2 outlet stream was eluted into the custom 3D printed alpha chamber equipped with a 600 mm^2^ Mirion Technologies PIPS detector.^5^ The alpha chamber was disconnected from the system, then connected to an Ortec 428 bias supply at +40 V with associated Ortec electronics including a 142 preamplifier, 855 dual spec amplifier, and a 927 Aspec MCA. Vacuum was applied for approximately 45 min to dry the sample using an Ortec ALPHA-MINI-PPS portable vacuum pump, then an alpha spectrum was collected over an extended counting interval up to 24 h to measure the ^238^Pu/^239,240^Pu activity ratio.^5^ In all cases, Pu alpha spectra closely resembled those previously published.^5^ Pu isotope ratios were measured by integrating counts in a narrow region of interest (ROI) around the centroid of each peak. The channel width of this ROI was maintained as equal for both peaks in each spectrum.

The Amptek X-123 CdTe detector was calibrated for efficiency and energy using ^109^Cd, ^123m^Te, ^152^Eu, ^155^Eu and ^237^U sources. UV–vis spectra were analyzed for U concentration using the characteristic U absorbances (414, 404, and 424 nm) when in solutions above 100 ppm with a 10 cm-long flow cell (SMA-Z-100 uvol, FIAlab Instruments) or using the U-PAR complex absorbance at 530 nm when below 10 ppm U with the 1 cm-long flow cell. The UV–vis spectrometer was calibrated using U ICP-MS standards from 100–500 ppm and 0.5–10 ppm with and without PAR solution, as appropriate. The PIPS alpha spectrometer was calibrated for energy using a mixed actinide source placed inside the custom alpha chamber. Each channel of the peristaltic pump was calibrated by weighing water collected at set flow rates prior to eluting samples. These calibrations were well parallelized, requiring a total of 2 h before the system was ready for sample introduction. Separation chemistry typically required less than an hour to reach steady state and then deliver sufficient U and Pu to the instrumentation for analysis. Radiometric acquisitions were constrained between 16–24 h for these experiments. Except for alpha spectrometry electronics, the assembled system had a footprint of approximately 2.5 by 3 feet, small enough to fit within a single vent hood, as depicted in Figure S2.

## 3 Results

### 3.1 Steady State and Flow Rate Studies

Similar to previous work,^19^ Figure 3a shows 10 min of elution was sufficient to achieve steady state operation at a flow rate of 10 μL min^–1^ for a single extraction. Slight improvement in U yield might be observed at 20 min but was not considered significant. The extrapolated steady state time for the *n* = 1 Extraction tower was approximated as 30 min, based on its two extraction membranes and approximately 135 μL of dead volume at a flow rate of 10 μL min^–1^. As expected, increasing flow rate is shown to negatively affect both U and Pu extraction yields in Figure 3b. A flow rate of 10 μL min^–1^ was used for the rest of this study to minimize analysis time, as the volume of the fully integrated system is several hundred microliters.

**Figure 3:**
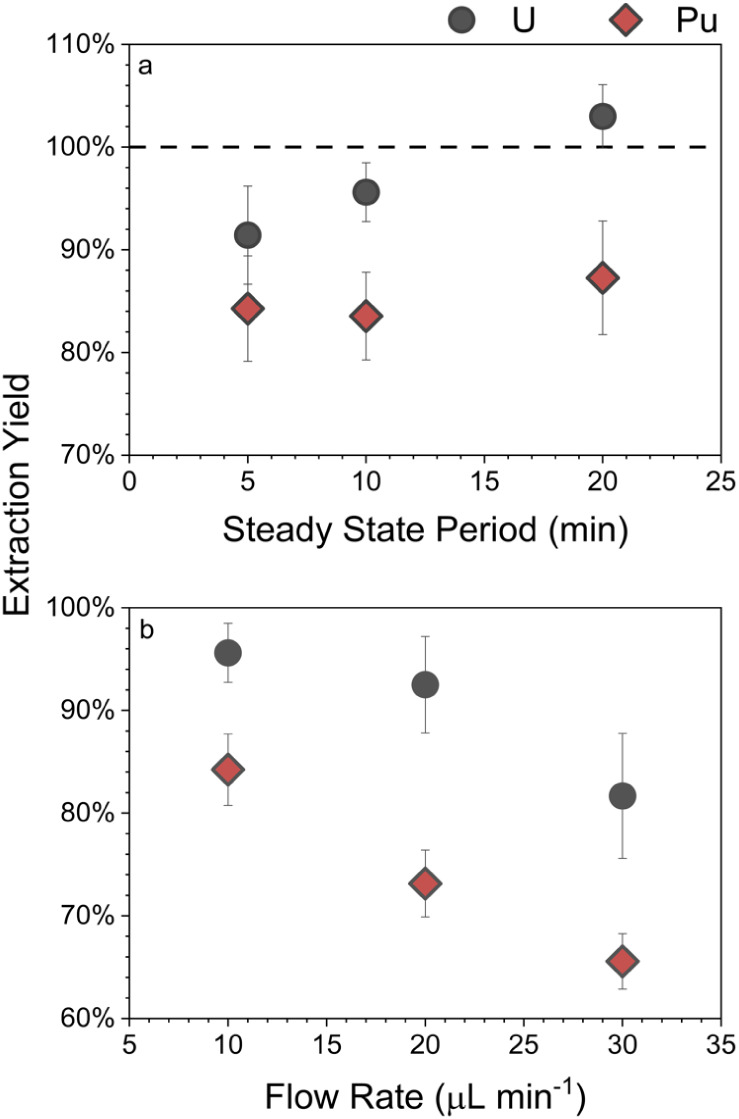
Steady state and flow rate studies. (a) U­(VI) (gray circles) and Pu­(IV) (red diamonds) extraction yields as a function of steady state time for a single extraction at a flow rate of 10 μL min^–1^. (b) U­(VI) (gray circles) and Pu­(IV) (red diamonds) extraction yields as a function of flow rate at a steady state time of 10 min. Feed solutions were 3 M HNO_3_ and strip solutions were 0.3 M HNO_3_. Error bars represent 1σ uncertainties.

### 3.2 Environmental Matrix Study

Figure 4 compares the extraction yields of U and Pu from the *n* = 1 Extraction Tower across the different environmental matrices tested. U is expected to elute into the S1 outlet stream, which requires U­(VI) to extract across both sequential TBP membranes. U yields are observed to be high across all tested environmental matrices, with a minor reduction in yield from the seawater matrix. This indicates that the U chemistry is extremely robust against sample matrix, which is ideal for an analysis which would be applied to a range of different environmental samples.

**Figure 4:**
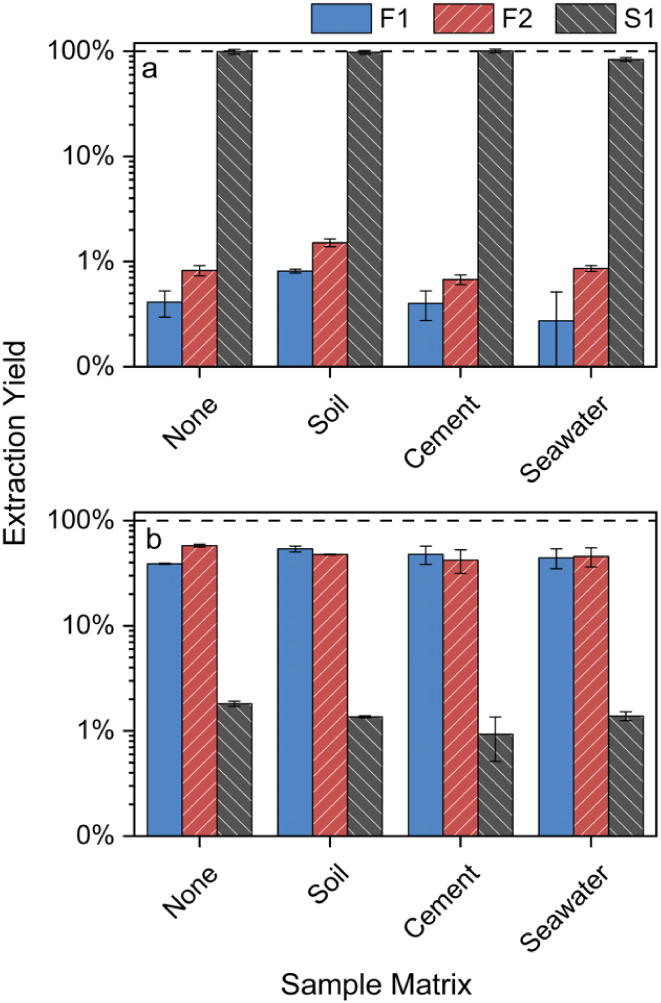
U and Pu yields in surrogate debris matrices. Extraction yields of U­(VI) (a) and Pu­(III,IV) (b) are reported for each of the environmental matrices tested. The F1 outlet stream (solid blue bars) is expected to carry most actinides, FPs, and MEs, the F2 outlet stream (red bars with diagonal hatch lines trending upward left to right) is expected to carry Pu­(III), and the S1 outlet stream (gray bars with diagonal hatch lines trending downward left to right) is expected to carry U­(VI). Feed solutions were 3 M HNO_3_ in three different matrices, the first strip solution was 0.3 M HNO_3_ 1 mM ascorbic acid 5 mM sulfamic acid, and the second strip solution was 0.3 M HNO_3_.

Pu is expected to elute into the F2 outlet stream. This requires Pu to extract across the first TBP membrane as Pu­(IV), reduce to Pu­(III), then fail to extract into the second TBP membrane. Initially, this process was inconsistent; the reduction of Pu­(IV) to (III) by 1 mM ascorbic acid (Figure 2, stream 3) failed across approximately 25% of all replicates performed with the FS-SLM platform without sulfamic acid present. However, the same reduction was successful in eight consecutive replicates performed in batch. Failures were identified in the FS-SLM platform by Pu eluting with high yield through the S1 outlet, which would require extraction across both TBP membranes as Pu­(IV). After a thorough investigation, it was identified that the addition of 5 mM sulfamic acid to the reducing stream (Figure 2, stream 3) resulted in consistent Pu reduction in the FS-SLM platform, as confirmed by all eight extractions reported in Figure 4b producing low Pu­(IV) yields in the S1 outlet stream. The initial reduction inconsistency was only identified when using the *n* = 1 Extraction Tower, not in batch. This is likely due to the mixing stage within the Extraction Tower, where the reducing stream (Figure 2, stream 3) is mixed with a low flow rate of 13 M HNO_3_ prior to the second extraction membrane. It seems likely that the addition of sulfamic acid prior to this mixing stage protects ascorbic acid against oxidation by HNO_3_, as autocatalyzed by HNO_2_.^26,27^ The decontamination study and application of the fully integrated system were performed prior to the implementation of sulfamic acid to the reducing stream. The addition of sulfamic acid is not expected to have a significant impact on the results of those experiments, aside from the improved consistency of Pu reduction.

The Pu yields reported in Figure 4b show that approximately half of all Pu elutes through the F1 outlet stream, resulting in an extraction yield of approximately 50% across the first TBP membrane even when no matrix was present. This is significantly lower than the approximately 85% yield reported from Figure 3a; the clear difference between these two experimental conditions is the inclusion of 1 mM ascorbic acid and 5 mM sulfamic acid in the stripping solution used only for the *n* = 1 Extraction Tower. This observation suggests that some ascorbic acid from the stripping stream may transfer across the first TBP membrane, stabilizing Pu­(III) and reducing yield. Pu yields varied between 40–60% across the duplicate cement and seawater extractions, resulting in larger uncertainties of their weighted averages seen in Figure 4b and indicating that yields may vary between each extraction. Despite these challenges, Pu yields in the F2 outlet stream were approximately 40–60% across each of the environmental matrices tested, which results in a sufficient count rate for Pu isotopic analysis.

### 3.3 FP and Np Decontamination Study

Short-lived FPs and actinides are among the radionuclides with the highest activities in postdetonation nuclear debris. The decontamination study was designed to measure the yield of some short-lived FPs and actinides to determine if their presence may have an impact on the ability to measure U and Pu isotopics as intended. Figure 5 reports the extraction yields of several short-lived FPs and Np measured in triplicate. These extractions did not include the addition of 5 mM sulfamic acid to the reducing strip and weren’t tested against the environmental matrices.

**Figure 5:**
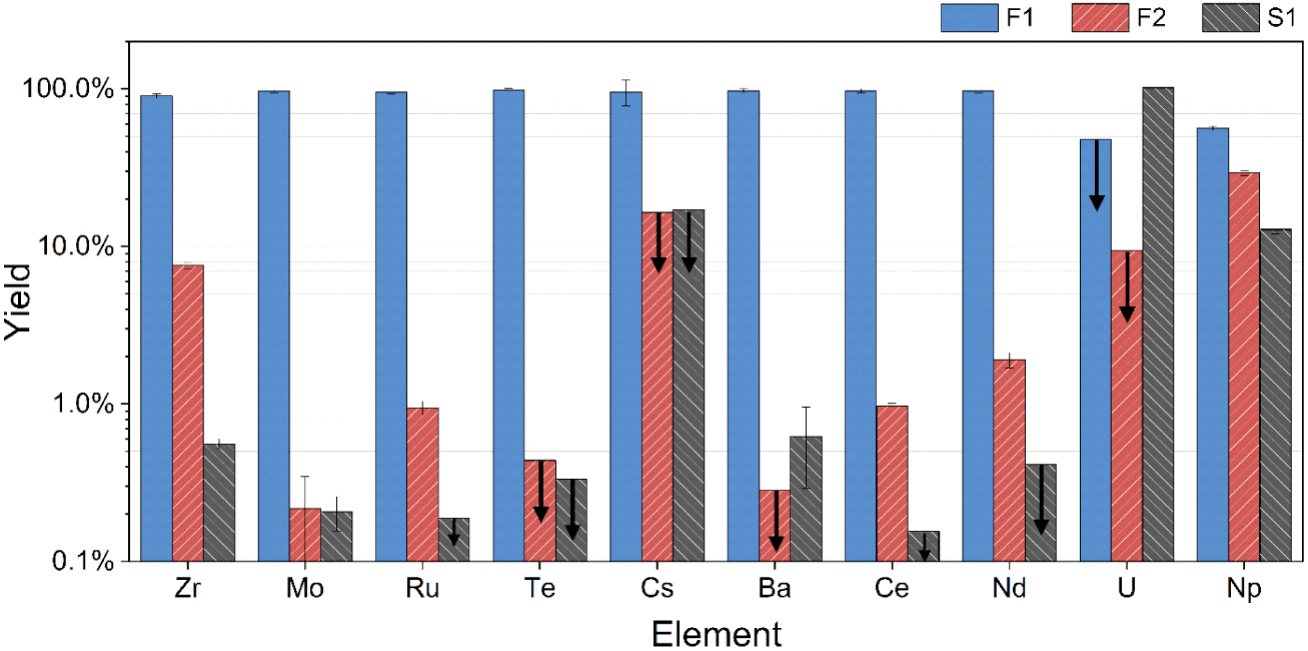
FP and Np decontamination study. Yields of several FP elements, U­(VI), and Np measured using gamma spectrometry from the *n* = 1 Extraction Tower. The F1 outlet stream (solid blue bars) is expected to carry most actinides, FPs, and MEs, the F2 outlet stream (red bars with diagonal hatch lines trending upward left to right) is expected to carry Pu­(III), and the S1 outlet stream (gray bars with diagonal hatch lines trending downward left to right) is expected to carry U­(VI). Feed solutions were 3 M HNO_3_, the first strip solution was 0.3 M HNO_3_ 1 mM ascorbic acid, and the second strip solution was 0.3 M HNO_3_. Mean values represent the weighted average of triplicate measurements and error bars represent 1σ uncertainties of the greater of their internal or external variance. Arrows represent upper limits by gamma spectrometry, driven by the high activity of ^239^Np present in each stream.

Np was found to partially extract in all three outlet streams; this is a serious concern as large activities of ^239^Np could potentially interfere with the downstream isotopic measurements of both U and Pu. ^239^Np has several photopeaks which could interfere with ^237^U quantification by gamma spectrometry and high dead times resulting from excessive beta activity could result in extremely long acquisition times for Pu analysis by alpha spectrometry. Np­(V) is known to disproportionate to Np­(IV) and Np­(VI) in nitric acid solutions^28^ and no specific effort to control the Np oxidation state was employed. The addition of a redox agent to stabilize Np­(V) in the sample feed solution may prevent this disproportionation and reduce its potential disruption to the U and Pu isotopic analyses. This has been shown to be successful for a single FS-SLM extraction in separate work,^29^ indicating promise for development into the U and Pu scheme described here. Generally, all upper limits in Figure 5 are constrained by the Compton backgrounds generated from the high activity of ^239^Np in the counting sample.

Otherwise, FP yields were observed to behave as expected. All FPs except for Zr were measured within uncertainty of 100% yield in the F1 outlet stream. These FPs were measured at 1% or less in the F2 and S1 outlet streams. Zr is known to weakly extract into 30% TBP from nitric acid media,^30–32^ which was observed here with 8% Zr yield in the F2 outlet stream. These results indicate that short-lived FPs are not expected to interfere with the gamma spectrometry measurement of ^237^U or the alpha spectrometry measurement of ^238,239,240^Pu following extraction.

### 3.4 Benchmarking the Fully Integrated System

Each component from our previous works^5,6,19^ was combined into the fully integrated system described by the schematic in Figure 2. Four of the samples prepared to benchmark this system had U concentrations of 250 ppm. This is much higher than the concentration of U in environmental samples; this high concentration is a direct limitation of the low ^237^U specific activity based on the production method used.^6^ These four samples did not require mixing the U outlet stream with PAR prior to UV–vis analysis. The fifth benchmarking sample contained 4 ppm U without environmental matrix, which required PAR mixing for UV–vis analysis and resulted in significantly higher counting uncertainties due to low ^237^U activity. The inclusion of sample matrix is not expected to impact the performance of PAR for determining U concentration, since the S1 outlet stream receives two selective extractions prior to analysis. These extractions did not utilize the addition of 5 mM sulfamic acid to the reducing stream, which may have impacted Pu yields resulting in larger counting uncertainties.

As shown in Figure 6 and tabulated in Table S3, the U and Pu isotopics measured with the fully integrated system were consistent with the standardized values in all tested conditions within 2σ uncertainty. These results indicate excellent ability to separate then quantify U and Pu isotopics from surrogate fission samples in environmental matrices using the fieldable platform. Consistency between the 4 and 250 ppm U samples indicates that this measurement can be applied across a range of U concentrations with the optional use of PAR. Previous work indicates that U concentrations as low as 1 ppm may also be measured accurately.^6^ These U concentrations remain relevant to postdetonation forensic efforts, but improving sensitivity is likely necessary to measure the range of environmental U levels in various environmental matrices.

**Figure 6:**
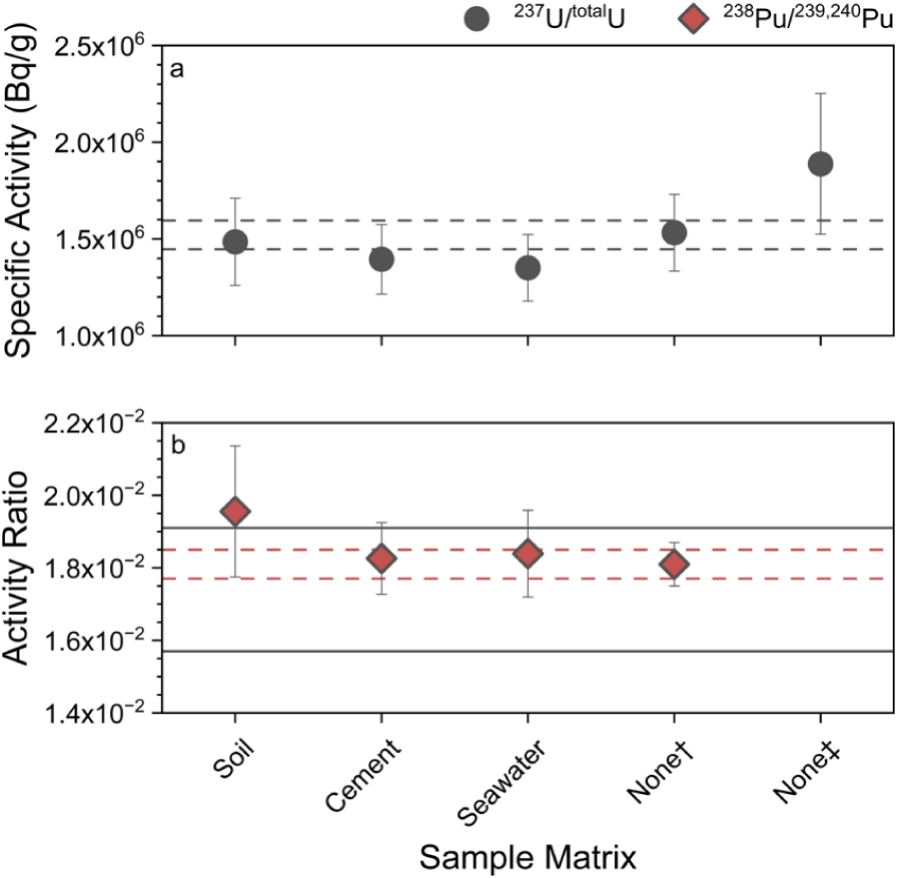
Benchmarked U and Pu isotopics. Measured U­(VI) (a) and Pu­(III,IV) (b) isotopics benchmarked against their standardized values. Feed solutions were 3 M HNO_3_ containing three different matrices, the first strip solution was 0.3 M HNO_3_ 1 mM ascorbic acid, and the second strip solution was 0.3 M HNO_3_. The range of 2σ uncertainty of certified values and standardized values are represented by the horizontal solid and dashed lines, respectively; error bars represent the 2σ uncertainty of the measured value. **^†^** 250 ppm U, ^‡^4 ppm U.

Because the individual activities of the reported Pu isotopes are measured simultaneously using the same alpha spectrometer, measurement uncertainty only includes counting uncertainty, varying between 2.7–4.6% between samples at 1σ. For U, reported uncertainties include counting uncertainty, uncertainty from the CdTe calibration, uncertainty from the UV–vis calibration, uncertainty of the peristaltic pump flow rates when mixing the U stream with the PAR solution, and a measured uncertainty for the volume of the CdTe detector loop. The uncertainties reported here vary between 6.3–9.6% at 1σ, with the highest uncertainty associated with the low activity of ^237^U in the 4 ppm U sample. The uncertainty budgets related to U isotopic analysis are discussed in detail in a previous publication.^6^

## 4 Conclusions

This work expands significantly upon previous developments of a fieldable microfluidic platform for the separation and assay of U and Pu to support rapid postdetonation debris analysis.^5,6,19,20^ The platform is intended to have a small footprint and utilize exclusively fieldable equipment and materials, such that it may be deployed in the event of a nuclear detonation. New developments include the Extraction Tower, capable of performing multiple sequential extractions, and investigating how the presence of short-lived FPs, Np, and environmental matrices affect the extraction of U and Pu when present in relevant quantities. Generally, U yields remain high under all conditions tested, while Pu yields are sufficient for the isotopic analyses performed. This work also integrated each of the developed components to separate U and Pu from samples containing environmental matrices and short-lived FPs, then performing isotopics assays to report the ^237^U/^total^U specific activity and ^238^Pu/^239,240^Pu activity ratio benchmarked within 2σ uncertainty of standardized values.

Typical analysis timelines used in this work included 2 h for setup and calibration, 1 h for chemistry and product collection, and 16–24 h for radiometric acquisition. The platform is small, less than 2.5 by 3 feet with most parts assembled, and as a microfluidic platform total reagent needs are minimal by volume and mass. These properties are advantageous for fieldable analysis efforts.

## Supplementary Material



## Notes

Reagents and materials; tabulated elemental concentrations in the environmental matrices as prepared for extraction; design of the Initiator, Propagator, and Terminator modules; tabulated U and Pu measurements from the fully integrated system; supporting offline gamma spectrometry, mass spectrometry, alpha spectrometry, and LSC measurements (PDF)
