# Supplementary material for: Fieldable Microfluidic Platform for Separation and Assay of U and Pu from Fission Samples in Environmental Matrices
Source: Ind Eng Chem Res. 2026 Jul 6;65(27):14566–73. doi: 10.1021/acs.iecr.6c01280 (PMC13383627; doi:10.1021/acs.iecr.6c01280)
Supplement: Supplementary file 1 [file ie6c01280_si_001.pdf]

Supporting Information for Publication

Fieldable Microfluidic Platform for Separation and Assay of U and Pu from Fission Samples in Environmental Matrices

Kevin J. Glennon[a]*, Hector F. Valdovinos[a], Jake A. Bence[a], Tashi Parsons-Davis[a], Narek Gharibyan[a], Jennifer A. Shusterman[a]

[a]Nuclear and Chemical Sciences Division, Lawrence Livermore National Laboratory, Livermore, CA 94550, USA

*Corresponding Author, glennon5@llnl.gov

Environmental Matrices

Samples containing environmental matrices were screened by ICP-MS for metal and other elemental concentrations as prepared for extraction. Soil refers to the prepared New Jersey Soil (SRM 2706) environmental matrix, cement refers to the prepared Portland Cement (SRM 635a) environmental matrix, and seawater refers to the prepared Atlantic Seawater (working standing obtained from Ocean Scientific International Ltd) environmental matrix.

Aliquots of samples and process blanks were analyzed in duplicate both with a Thermo Scientific Element XR magnetic sector field inductively coupled plasma mass spectrometer (SF-ICP-MS) and a Thermo Scientific iCAP triple quadrupole inductively coupled plasma mass spectrometer (TQ-ICP-MS). SF-ICP-MS was employed due to its ability to attain high mass resolution and detect analytes without interference from various polyatomic ions. Depending on the analyte, this instrument was operated in low, medium, or high-resolution modes ($m/\Delta m = 300$, 4000, and 10,000 respectively). TQ-ICP-MS was employed in either no–gas (NG) mode or using helium, oxygen, or hydrogen in the second

quadrupole, reducing isobaric and polyatomic interferences to arrive at a selective determination of analytes.

Standards and samples were prepared gravimetrically. Fully quantitative analyses using a linear calibration curve based on certified external standards were performed. An internal standard was used to determine corrections for instrument drift. All reported uncertainties incorporate statistical and systematic components. A method detection limit was calculated as the standard deviation of the analytical results of three process blanks for each sample, multiplied by a coverage factor, k. The detection limit was calculated at the 99% CI (k = 9.925, two-tailed t-statistic for n =3). Analytes below the detection limit are not reported.

Table S1. Tabulated elemental concentrations reported with 1σ uncertainty by the ICP-MS screening of environmental samples as prepared for extraction.

Element	Soil (μg/g)	Relative Uncertainty	Cement (μg/g)	Relative Uncertainty	Seawater (μg/g)	Relative Uncertainty
Li	1.31E+00	13.7%			1.82E-01	0.4%
Be	7.70E-02	57.8%				
B					5.51E+00	0.3%
Na	2.13E+02	1.0%	1.18E+02	1.2%	7.36E+03	0.7%
Mg	1.79E+02	0.5%	6.34E+02	0.7%	1.17E+03	0.4%
Al	1.99E+03	1.2%	9.39E+02	1.0%	1.13E+00	0.4%
Si	9.70E+00	49.5%	9.00E+00	34.4%	3.34E+00	0.4%
P			1.04E+01	2.4%	2.90E-02	0.6%
S	1.25E+02	1.0%	2.62E+01	1.4%	1.03E+03	0.3%
K	2.51E+02	0.7%	1.60E+02	1.4%	1.54E+02	0.5%
Ca	3.70E+02	1.3%	8.26E+03	0.4%	3.93E+02	0.8%
Sc	3.07E-01	4.4%				
Ti	9.78E+01	0.8%	4.94E+01	0.9%		
V	3.23E+00	1.1%				
Cr	2.55E+00	1.0%	1.84E+00	0.5%		
Mn	1.74E+01	0.9%	2.42E+01	0.6%	7.96E-03	0.3%
Fe	1.53E+03	1.0%	5.78E+02	0.6%	3.51E-01	0.3%
Co	3.95E-01	0.6%				
Ni	1.50E+00	1.0%			5.39E-03	2.6%
Cu	6.69E+00	1.0%			1.59E-02	0.9%
Zn	8.85E+00	0.9%	5.52E+00	1.1%	7.09E-02	0.4%
Ga	6.83E-01	1.8%				
As	8.70E-01	2.2%			1.47E-03	7.8%
Rb	2.63E+00	0.7%			1.13E-01	0.2%
Sr	4.23E+00	1.1%	2.82E+01	0.4%	7.55E+00	0.2%
Zr	2.56E+00	0.8%				
Nb	2.53E-01	0.3%				
Mo	7.97E-02	2.6%				
Cd	1.97E-02	10.7%			9.24E-04	2.9%
Sn	2.20E+00	0.8%				
Sb	9.04E+00	0.8%				
Cs	8.45E-02	2.0%			2.99E-03	1.8%
Ba	1.96E+01	0.9%	7.86E+00	1.1%	2.18E-01	0.3%
La	1.00E+00	0.5%				
Ce	2.15E+00	0.5%				
Pr	2.42E-01	1.2%				
Nd	9.61E-01	1.1%				
Sm	1.84E-01	1.3%				
Eu	3.84E-02	2.6%				
Gd	1.38E-01	2.1%				
Tb	2.13E-02	1.4%				

Dy	1.20E-01	2.0%				
Ho	2.21E-02	2.7%				
Er	6.39E-02	2.3%				
Tm	8.74E-03	3.5%				
Lu	7.99E-03	2.8%				
W	2.25E-02	2.1%				
Tl	1.48E-02	2.8%				
Pb	4.20E+01	0.8%			1.07E-02	1.0%
Bi	1.02E-02	3.0%				
Th	2.63E-01	0.5%				
U	7.19E-02	1.5%			3.46E-03	11.7%

Initiator, Propagator, and Terminator Modules

Each FS-SLM extraction module was designed using FreeCAD 1.0 and 3D printed using a Formlabs Form 4 SLA printer, depicted in Figure S1. SLA 3D printers can produce much finer surface features than internal ones. In this work, the smallest diameter internal channel that could be reliably 3D printed was approximately 1.2 mm; however, surface features as small as 50 or 100 μm were consistently achievable. To take advantage of this, each FS-SLM module was designed to minimize internal features while utilizing half-extraction channels printed on their surface. Then, an 80 μm thick PTFE membrane can be placed on top of these surface half-channels, and two modules bolted together to produce a complete extraction system where aqueous solutions flow across both sides of a membrane soaked in the selective extractant.

Each extraction channel is given the same cross section and follows the same serpentine path; the channels were designed to maximize the surface-to-volume ratio within the space available. After placing the extractant-soaked PTFE membranes between extraction channels, the modules were bolted together using 4x 8-32 bolts with nuts and washers. The volumes of each feature in the FS-SLM modules are tabulated in Table S2; when connected to the fully integrated system, total system volumes include significant lengths of tubing and instrumentation, which results in even larger volumes.

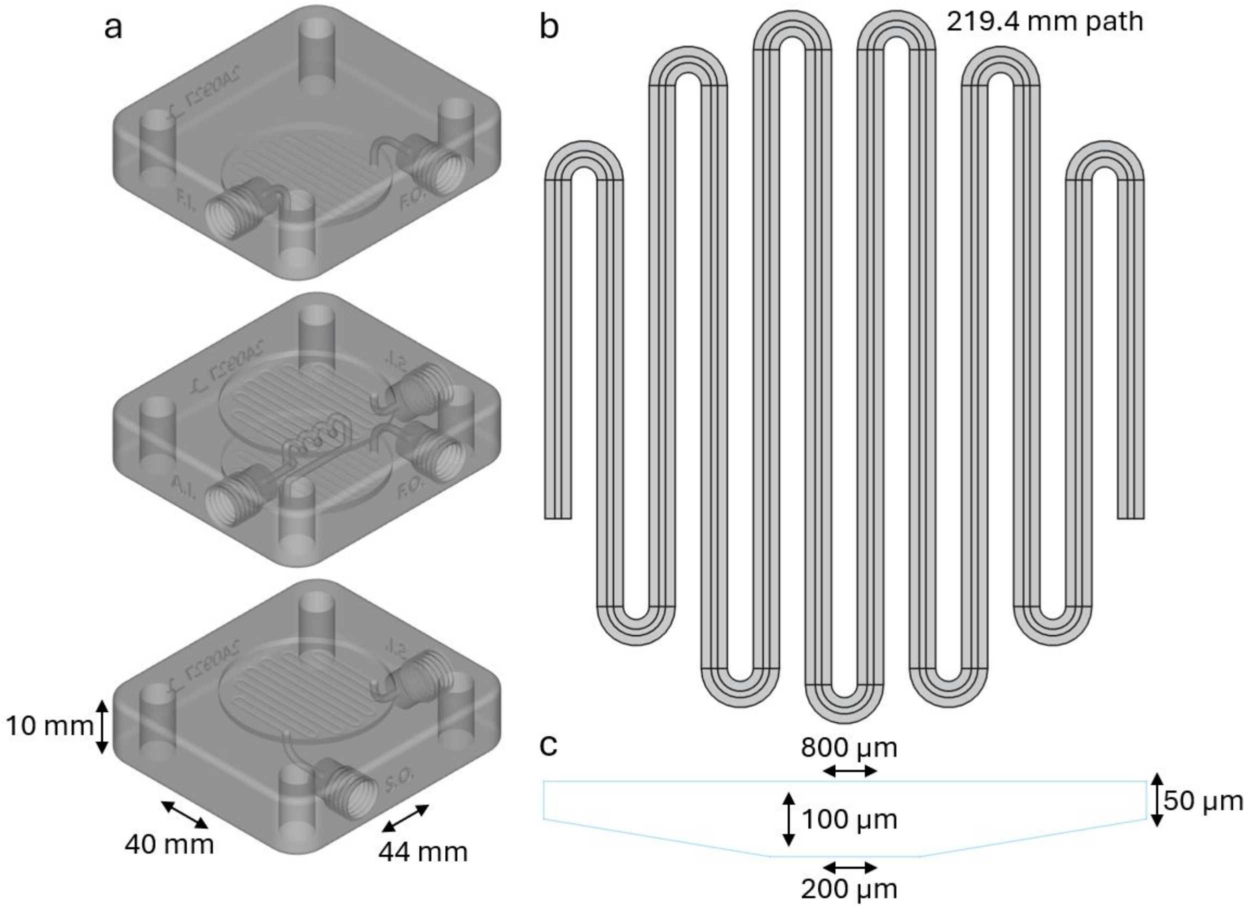

Figure S1. Description of the FS-SLM modules. a) Initiator, Propagator, and Terminator modules from top to bottom, using transparent shading to highlight internal features. b) The half-extraction channel path present on each FS-SLM surface. c) The cross section of each half-extraction channel.

Table S2. Enclosed channel and extraction channel volumes of each FS-SLM module.

Module	Inlet and Outlet Volume (μL)	Extraction Channel Volume (μL)	Helix Mixer Volume (μL)	Total Module Volume (μL)
Initiator	28.1	14.3	-	42.4
Propagator	23.5	28.6	61.9	114.0
Terminator	21.5	14.3	-	35.8
Total	73.1	57.2	61.9	192.2

Fully Integrated System

The measured $^{237}U/^{total}U$ and $^{238}Pu/^{239,240}Pu$ ratios are visualized as Figure 6 in the main text and tabulated here as Table S3. From the main-text, the standardized $^{237}U/^{total}U$ specific activity was (1.521 ± 0.037)x10^{6} Bq/g, the certified $^{238}Pu/^{239,240}Pu$ activity ratio was (1.74 ± 0.17)x10^{-2}, and the standardized Pu activity ratio was (1.81 ± 0.02)x10^{-2} as of February 2023 when the experiments were performed. An image of the fully assembled integrated measurement platform, except for stand-alone alpha spectrometry electronics, is provided as Figure S2.

Table S3. Tabulated measurements of the U and Pu ratios from the fully integrated system.

Matrix	U Concentration (ppm)	$^{238}Pu/^{239,240}Pu$ Activity Ratio	Relative Uncertainty	$^{237}U/^{total}U$ Specific Activity	Relative Uncertainty
Soil	250	1.96E-02	4.6%	1.49E+06	7.6%
Cement	250	1.83E-02	2.7%	1.40E+06	6.4%
Seawater	250	1.84E-02	3.3%	1.35E+06	6.3%
None	250	1.81E-02	1.7%	1.53E+06	6.5%
None	4			1.89E+06	9.6%

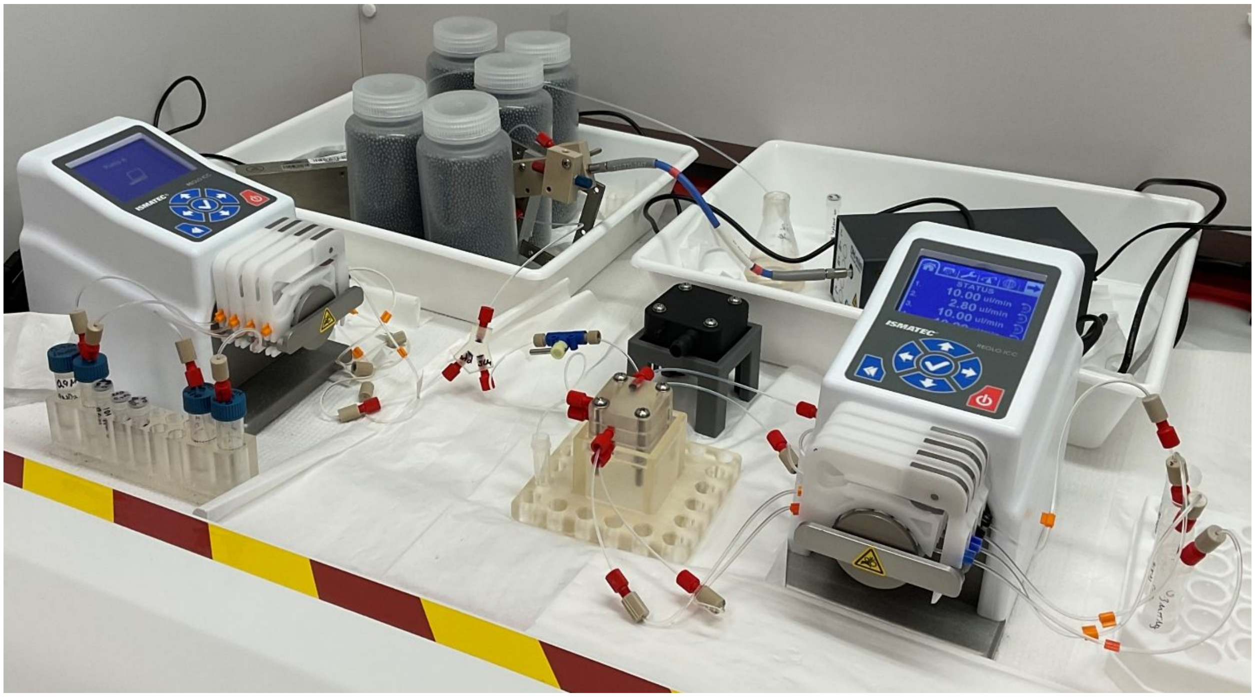

Figure S2. The fully integrated FS-SLM platform. Image includes all chemistry and instrumentation required except alpha spectrometry electronics. Approximately 2.5 by 3 feet.

Offline Measurements

Offline gamma spectrometry and mass spectrometry were used to determine the known $^{237}U/^{total}U$ specific activity prior to making each surrogate fission sample. Offline gamma spectrometry was also used for measuring extraction yields of ^{239}Np and short-lived FPs. Offline liquid scintillation counting (LSC) was used to measure extraction yields of U and Pu in various studies. Offline alpha spectrometry was used to determine the standardized $^{238}Pu/^{239,240}Pu$ activity ratio at a lower uncertainty than provided in the CRM 138 certificate.

For offline gamma spectrometry, counting samples were prepared by weighing 25 – 100 μL sample aliquots into a standardized geometry vial and diluting up to 10 mL with H_2O. These samples were counted for 24 hours at standardized distances from well-calibrated

HPGe detectors to determine the activity of each gamma-emitting radionuclide of interest. Peak fitting and efficiency corrections were achieved using the in-house software Gamanal. For offline alpha spectrometry, a small volume < 20 μL of the separated CRM 138 Pu source was stippled onto an aluminum planchette, then evaporated to dryness on a hotplate. The dry alpha source was placed in a standard ORTEC Alpha Mega alpha chamber and an alpha spectrum was acquired over 12 hours. Pu isotope ratios were measured by integrating counts in a narrow region of interest (ROI) around the centroid of each peak. The channel width of this ROI was maintained as equal for both peaks in each spectrum. Mass spectrometry was performed using a ThermoScientific iCAP-Q quadrupole ICP-MS, using a mass bias solution (CRM U010) and a QC solution (CRM U005A) to ensure accurate $^{total}U$ quantification. LSC was performed by weighing and aliquoting 100 μL of each stream into 10 mL of Ultima Gold AB scintillation cocktail and assaying with a Perkin Elmer Tri-Carb 3110TR, assuming 100% scintillation efficiency for alpha decay.
